# Characterization of plasma metal profiles in Alzheimer’s disease using multivariate statistical analysis

**DOI:** 10.1371/journal.pone.0178271

**Published:** 2017-07-18

**Authors:** Chunmei Guan, Rui Dang, Yu Cui, Liyan Liu, Xiaobei Chen, Xiaoyu Wang, Jingli Zhu, Donggang Li, Junwei Li, Decai Wang

**Affiliations:** 1 Department of Nutrition and Food Hygiene, Public Health College, Harbin Medical University, Harbin, Heilongjiang, China; 2 Hobo college of Xinjiang Medical University, Xinjiang, Kelamayi, China; 3 Department of Social Medicine, Public Health College, Harbin Medical University, Harbin, Heilongjiang, China; 4 Institute of Quality Supervision and Detection, Harbin, Heilongjiang, China; ITALY

## Abstract

The exact cause of Alzheimer’s disease (AD) and the role of metals in its etiology remain unclear. We have used an analytical approach, based on inductively coupled plasma mass spectrometry coupled with multivariate statistical analysis, to study the profiles of a wide range of metals in AD patients and healthy controls. AD cannot be cured and the lack of sensitive biomarkers that can be used in the early stages of the disease may contribute to this treatment failure. In the present study, we measured plasma levels of amyloid-β_1–42_(0.142±0.029μg/L)and furin(2.292±1.54μg/L), together with those of the metalloproteinases, insulin-degrading enzyme(1.459±1.14μg/L) and neprilysin(0.073±0.015μg/L), in order to develop biomarkers for AD. Partial least squares discriminant analysis models were used to refine intergroup differences and we discovered that four metals(Mn, Al, Li, Cu) in peripheral blood were strongly associated with AD. Aberration in homeostasis of these metals may alter levels of proteinases, such as furin, which are associated with neurodegeneration in AD and can be a used as plasma-based biomarkers.

## Introduction

Alzheimer’s disease(AD) is an insidiously, irreversible and progressive age related neurodegenerative disease, and currently affects over 35 million individuals worldwide[1]. The accumulation of aggregated amyloid β (Aβ) in extracellular plaques and neuronal fibrillary tangles (NFTs) of phosphorylated τ are two major neuropathological hallmarks of AD and that neuropathological changes can produce cognitive decline, memory impairments, as well as psychobehavioral disturbances, and ultimately, it is fatal[2]. There is considerable knowledge concerning the underlying processes in AD, amyloid cascade hypothesis, calcium dysregulation, oxidative stress, and synaptic toxicity[3]. However, the precise changes in the brain that trigger the development of AD, and the order in which they occur, remain unknown.

The concentration of a metal in the periphery may be classified as deficient, adequate, or excessive depending on genetic and/or environmental factors[4]. The result of a deficient or excessive exposure however has to consider chronic or acute disease occurrence, such as AD. Metals present as labile ions are proposed to play a pivotal role in the onset of AD, however, they are not the only ones. Aging and a higher rate of oxidative metabolism in the brain are two major risk factor for metal ion homeostasis. In AD, metal ions induce soluble Aβ to aggregate and precipitate, as well as promote the formation of reactive oxygen species (ROS), which can be generated from molecular oxygen by irreversible change in the biomacromolecules, and lead to metal-evoked oxidative stress[5]. Several studies have investigated brain zinc levels positively correlated with levels of Aβ peptide, plaque numbers, and dementia severity in AD[6]. In contrast to zinc,serum aluminum concentration tends to increase in AD[7]. Neurodegeneration in AD by copper-induced toxicity is mediated by binding to Aβ protein, leading to Aβ aggregates, and copper became the pro-oxidant in this process[8]. Moreover, post-mortem examination clearly suggests that bioavailable metals are found in high concentrations in the amyloid plaques[9]. Microparticle-induced X-ray emission analysis demonstrates that there are elevations in zinc, copper and iron in the neuropil of AD patients[10]. Undoubtedly, all these demonstrate that a delicate balance of metal activity is necessary for prolonged neuronal functioning. Despite extensive understanding of each of these metals individually as they occur within the cell, an adequate explanation for their origins, interactions, and evolution as they pertain to AD is lacking.

Metallomics is an emerging scientific field that was proposed by Hiroki Haraguchi at the 2002 international symposium on Bio-trace Element held in Japan[11]. Metallomics comprises determining the identities of individual metal species and their concentration, and elucidates the relationships between metals, metalloproteinases and other metal-containing biomolecules. Here, we used for the first time a wide range of metals in complex with metalloproteinases to explore the effect of metal ions on the evolution of AD. In addition, Inductively coupled plasma mass spectrometry (ICP-MS) was utilized for metal quantification and validated in human plasma by means of a single preparation procedure for each sample. ICP-MS is an excellent tool for the direct or after-digestion determination of a wide range of trace elements in body fluids because of its greater sensitivity and fewer polyatomic interferences compared with other traditional techniques[12]. ELISA kit was highly specific, sensitive, and suitable for large sample analysis[13], it was used to determine levels of metalloproteinases. The present study focused on analysis of 21 elements in plasma samples, in order to understand whether the dysregulation of metals is involved in the etiology of AD, and is to establish potential biomarkers for early diagnosis.

## Methods

### Plasma samples

Plasma samples from 92 patients with a diagnosis of probable AD according to NINCDS-ADRDA(National Institute of Neurological and Communicative Disorders and Stroke and Alzheimer Disease and Related Disorders Association)criteria were provided by the Centre of Harbin Elderly Care Service, Heilongjiang Province, China. Patients with mild cognitive impairment were excluded from the study. Healthy controls (HCs, n = 161) had no clinical evidence of neurological or psychiatric disease. The local Clinical Research Ethics Committees approved the study. All subjects gave written informed consent, except for AD patients who could not give consent. In this case, surrogate consent was obtained from guardians. All participants were over 65 years old.

### Reagents and instruments

Nitric acid (purity 65%–68%, guaranteed reagent) and hydrogen peroxide (purity 30%, guaranteed reagent) were purchased from Kermel Chemical Reagent Co.,Ltd.(Tianjing, China).Standard mixing solution, aqueous tuning solution and internal standard mixing solution were obtained from Agilent Technologies Inc. (Santa Clara, CA, USA).ELISA kits for measuring levels of insulin-degrading enzyme (IDE), neprilysin, furin and Aβ_1–42_were purchased from Nanjing Jiancheng Bioengineering Institute (Shanghai, China). All solutions were prepared using metal-free water produced using a Milli-Q system(Merck Millipore Santa Clara, USA).

Twenty-one metallic elements(Li^7^, Mg^24^, Al^27^, Ca^42^, Ti^47^, V^51^, Cr^52^, Mn^55^, Fe^56^, Co^59^, Ni^60^, Cu^63^, Zn^66^, As^75^, Se^78^, Sr^88^, Mo^95^, Cd^111^, Ba^137^, Tl^205^ and Pb^208^) in plasma samples were assayed simultaneously using ICP-MS (ICP-MS7700x,Agilent Technologies Inc.). The samples, together with solutions of internal standards (scandium, germanium, rubidium, terbium and bismuth, all 0.1μg/L), were injected in a ratio of 1:1 through a T-branch pipe. An on-line reaction cell filled with helium (>99.999%) was used to eliminate polyatomic interference caused by compounds with similar mass-charge ratios. ICP-MS tuning solution was used and conditions were optimized as follows: radiofrequency power, 1550W; sampling depth, 7.9mm;carrier gas flow rate, 1.03L min^–1^,ω bias voltage, ‒100V; ω lens voltage,7.4Vand octopolebias voltage,‒8V.

A thermostated oven(Boxun Industry & Commerce Co.Ltd., Shanghai, China) was used for digestion of samples, which was carried out in Teflon tubes. All biochemical measurements on plasma samples were automated using a microplate reader(Tecan Group Ltd.,Männedorf, Switzerland) and performed in duplicate.

### Sample preparation

Plasma samples were collected in the morning after an 8 h fast and rapidly centrifuged at 860×*g* for 15 min. The supernatants were divided into aliquots in Eppendorf tubes and frozen at ‒80°C until analysis. After thawing, plasma samples (200 μL)were vortexed for 10 min and then treated with 6% nitric acid and hydrogen peroxide (300 μL). The samples were digested in a thermostated oven at 130°C for 2 h and then diluted to a volume of 5 mL using ultrapure water. The plasma samples were thus diluted 25-fold. The detection limits of the 21 metallic elements were in the range 0.001–33.159μg/L.

Solid-phase sandwich ELISA kits, based on monoclonal antibodies, were used to determine Aβ_1–42_, furin, IDE and neprilysin. Plasma samples were vortexed at 1000×*g* for 30min to remove particulates and all reagents were warmed to room temperature before the experiments. The kits were used in accordance with the manufacturer’s instructions and the minimum detectable concentration of analytes in the assays was estimated to be 0.1 ng/mL.

### Statistical analysis

The dataset was built using Epi3.1 software and statistical analysis was conducted using SPSS 13.0 software and SIMCA-P12.0. Data are presented as mean ± standard deviation(SD) ($\bar{\text{x}}\pm s$).A *p* value <0.05 was considered to be significant in all statistical analyses.

#### Demographic analysis

Two groups were defined in terms of main demographic characteristics and statistically compared using two independent-samples Student’s *t* tests or χ^2^ tests. A multi-factor logistic regression model was also used to explore the association between the prevalence of AD and various demographic characteristics.

#### Analysis of metal profiles

Statistical comparisons of metal concentrations between the two groups were carried out using the Mann-Whitney *U* test. Pattern recognition, using partial least squares-discrimination analysis (PLS-DA), was employed for further analysis of the multidimensional data. PLS-DA converts multidimensional data into a low- dimensional model by reducing the high number of variables and generating new components.

#### Biochemical analysis

Significant differences between two groups were determined using independent two-sample Student’s t-tests.

## Result

Comprehensive demographics, including mini-mental state exam, body mass index, age, sex, history of smoking, drinking, drug use, and the type of tableware/cooking vessels used are presented in Table 1. The only significant differences (*p* < 0.05) between AD patients and HCs were in AD score, drug use, and type of tableware/cooking vessels used. A logistic regression model was used to explore the association between the prevalence of AD and different demographic characteristics. The consumption of aluminum-containing drugs or frequent use of aluminum- containing cooking vessels seemed to be a risk factor for AD [(*p*<0.05, odds ratio (OR)>1].

**Table 1:** Demographic characteristic and logistic analysis of investigated groups($\bar{x}\pm s$).

Classification	AD	HC	*p* value	*p* value ^a^	OR(95%CI)^a^
**N (n/total)**	92/253	161/253	—	—	—
**MMSE**	8.518±2.95	25.085±2.57	<0.05^b^	—	—
**BMI**	23.15±5.51	23.47±5.20	0.653^b^	0.405	0.977(0.923,1.032)
**Age, years**	76.59±7.31	77.84±8.79	0.226^b^	0.428	0.851(0.578,1.269)
**Sex** **(male/female)**	34/58	71/90	0.267^c^	0.304	1.408(0.734,2.702)
**Smoking** **(yes/no)**	67/25	104/57	0.178^c^	0.540	0.795(0.383,1.653)
**Drinking** **(<1/week, ≥1/week)**	78/14	138/23	0.840^c^	0.504	1.134(0.785,1.638)
**Drugs** **(aluminum contained)**					
**(<1/week, ≥1/week)**	63/30	148/13	<0.05^c^	<0.05	1.519(1.183,1.948)
**Tableware** **(aluminum contained)**					
**(<1/week, ≥1/week)**	44/48	123/28	<0.05^c^	<0.05	1.290(1.178,1.992)

MMSE: mini-mental state examination, total scores:30, mild cognitive impairment:13~23, moderate cognitive impairment:5~12, severe cognitive impairment:<5. CI: confidence intervals.—not detected.

^a^ logistic regression analysis.

^b^ 2-tailed T test.

^c^ Chi square test.

ICP-MS was used to determine 21 elements (lithium, magnesium, aluminum, calcium, titanium, vanadium, cadmium, manganese, iron, cobalt, nickel, copper, zinc, arsenic, selenium, strontium, molybdenum, cadmium, barium, thallium and plumbum) in plasma samples. The accuracy of the procedure for metal determination was verified by spike recovery tests and by using calibration samples prepared from the 21 metals. Repeatability of calibration samples was expressed as percentage bias and coefficient of variation. Percentage bias was in the range 95.49%–109.63% and coefficient of variation was in the range 0.38–4.57%, indicating that the method is suitable for metal determinations.

Metal concentrations in plasma samples from AD patients and HCs are summarized. The significance of differences in continuous variables between AD patients and HCs was determined using the Mann-Whitney *U* test. Levels of lithium, aluminum, manganese, iron, copper and zinc differed significantly between the two groups. Levels of lithium and manganese were very significantly decreased in AD patients (*p*<0.001) compared with the control group and levels of zinc were significantly reduced (*p*<0.05).Levels of aluminum, copper and iron, on the other hand, were significantly higher (*p*<0.001) in AD patients compared with HCs. Although the differences were not significant, higher levels of calcium, titanium, vanadium and cadmium were found in the AD group compared with the control group, whereas levels of magnesium, chromium, cobalt, selenium, strontium, molybdenum, barium and lead were lower.

**Fig 1:**
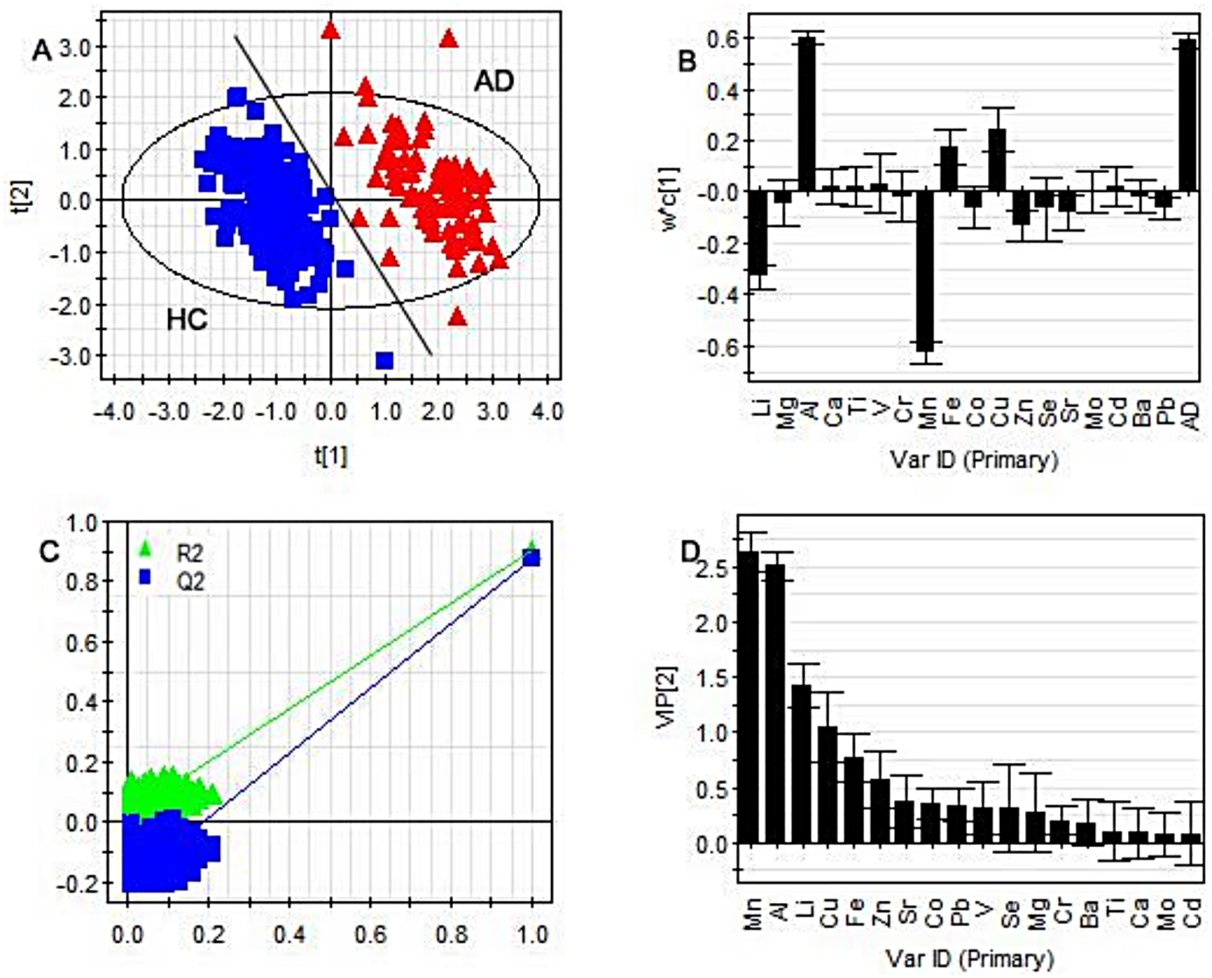
PLS-DA model of the ICP-MS data (excluding outliers) from AD and HC groups. (A)Scores plot, ■HC, ▲AD. (Only when the two sets of data shown in the model is completely separated, it can prove that the success of this model) (B) Loading plot. (The position of those metals located in the upper are prove that their patient group content is higher than the normal group. Otherwise, is lower than the normal group.) (C) Validation. (two lines represent Q2 and R2 were intersectanting which means the model work.) (D) Variable importance plot. (VIP could reflect the variable importance and identify potential biomarkers. The metal which VIP values >1.0 indicate that it is important for the development of AD).

PLS-DA, which is a multivariate classification method based on PLS, explains the maximum separation between defined class samples in a data set and is complementary to principal component analysis (PCA). PCA allows visual inspection of data, which facilitates detection of possible outliers [14], and was thus used for preliminary evaluation of data quality using concentrations of all metals (Ni, As and Tl were excluded because they were not detectable). Data for eight samples were discarded from further analysis because they were outside the 95% confidence interval. PLS-DA was applied to the same data set to sharpen the separation between data from AD patients and HCs. The PLS-DA scores plot(Fig 1A)discriminated unambiguously between samples from AD patients and those from HCs. Values of R2Y (0.890) and Q2Y (0.872) explained the variation in the response, Y, and represented the cross-validated results[15].

A loading plot (Fig 1B) allowed identification of potential discriminant biomarkers. The loading plot revealed that most of the metals tested contributed to the separation between the classes. This means that the respective contributions of individual components to the discrimination between the two groups can be readily obtained from the plot. Metals clustered on the right side of the plot were over-expressed in AD patients, while the others were reduced. Levels of aluminum, copper and iron were higher in AD patients and levels of manganese, lithium and zinc were lower.

In the validation plot (Fig 1C),the two lines representing Q_2_ and R_2_intersect, demonstrating the success of the PLS-DA model. The variable importance plot (VIP) reflects the importance of individual variables and identifies potential biomarkers. Using two criteria (VIP >1.0 and SD < mean value), we analyzed the set of metals for potential biomarkers capable of distinguishing between the AD and HC groups (Fig 1D). Manganese, aluminum, lithium and copper were identified as potential biomarkers, in agreement with the results of the loading plot.

**Fig 2:**
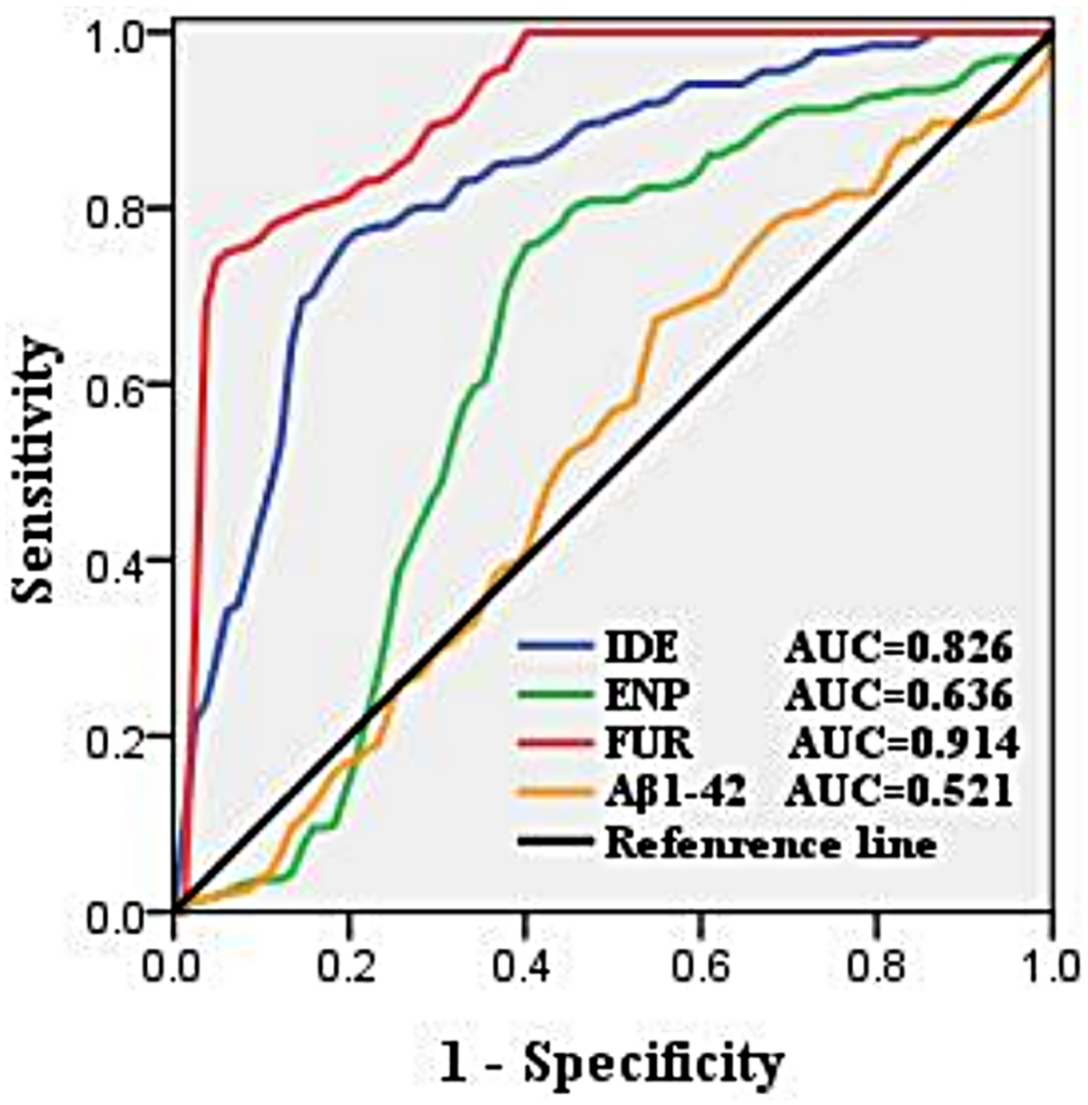
ROC analysis for discrimination of AD and control groups. ROC curve was a necessary tool for better interpretation of the results in the biomarker classification studies. Notably, ROC curves were empirical curves in the sensitivity and specificity space.

ELISA was used to study the effect of plasma metalloproteinases on Aβ_1–42_ aggregation in AD patients and HCs. IDE, neprilysin and furin were decreased in the AD group but Aβ_1–42_ was increased in the same individuals. Statistical analysis showed a significant difference in IDE and furin between the two groups (*p*<0.05). To refine this analysis, we constructed a receiver operating characteristic (ROC) curve, which is an essential tool for better interpretation of results in biomarker classification studies. Notably, ROC curves are empirical curves in the sensitivity and specificity space. We hoped to develop a clinical test with high specificity and, therefore, focused on partial AUCs with specificity of 80%–100%.Furin gave the best AUC (0.914), closely followed by IDE (0.826) (Fig 2).

## Discussion

**Fig 3:**
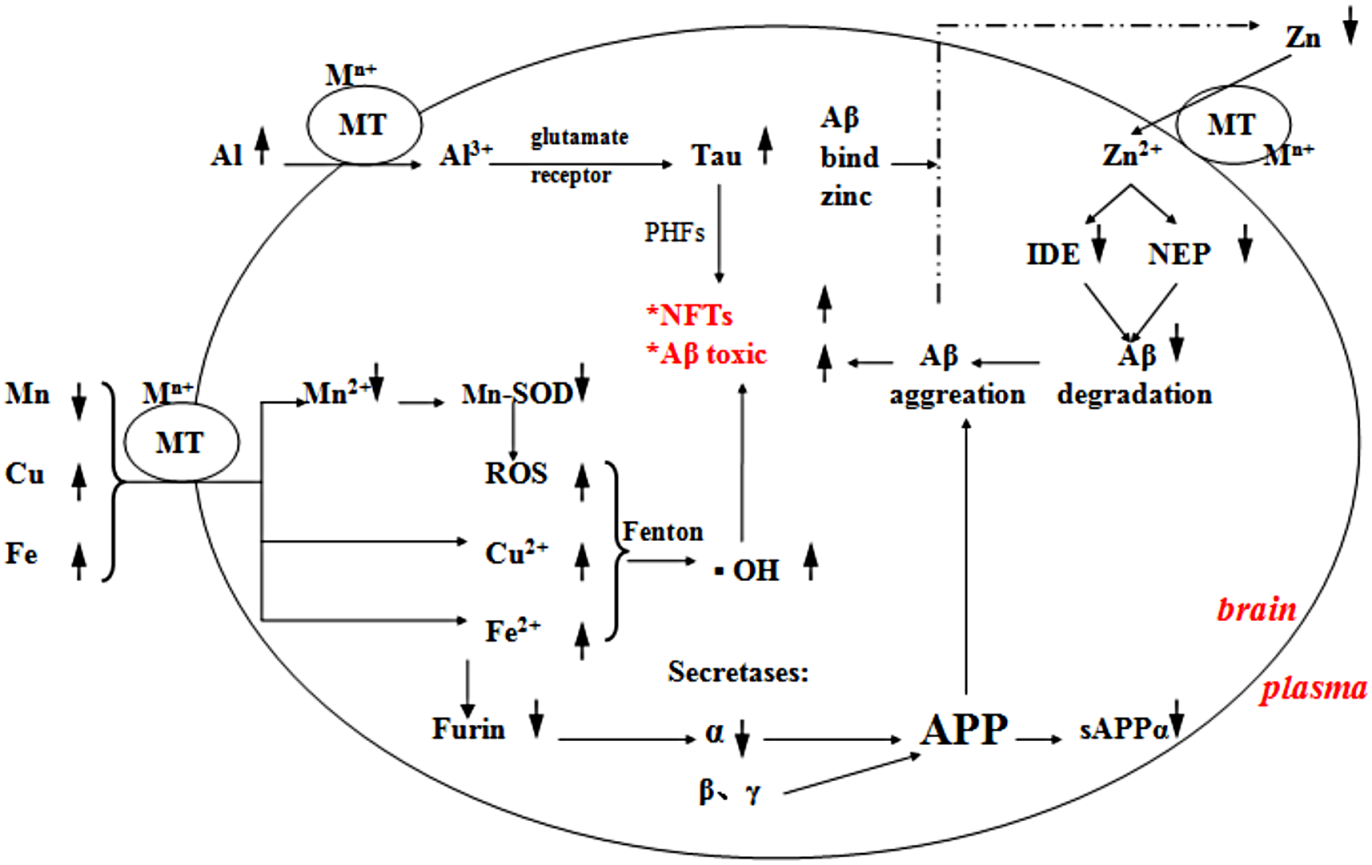
Characterization of plasma metal profiles in AD. The up and down arrows represent the measured indexes that were significantly increased or decreased in the AD group compared with the HC group. Inside the oval represents the brain, outside represents the plasma, MT represents metal transport and M^n+^ metal ions. The indexes, asterisks in the figure, are two major neuropathological changes in AD.

Two processes are linked to Aβ neurotoxicity or NFTs in AD patients. One of these is the induction of oxidative stress through ROS generation and the other is facilitation of Aβ aggregation or NFT formation, which results in weakened cell signaling and neuronal cell death. In the present study, we have refined our understanding of these complex processes (Fig 3).

The brain can regulate metabolism and transport of metal ions to maintain homeostasis but changes in levels of these metal ions in peripheral fluids may disrupt regulation in the brain and could be fundamentally involved in the pathogenesis of AD. Metal transporters transport metals across the blood-brain barrier (BBB) into the brain and it is clear that overload or deficiency of one metal is sufficient to induce a cascade of downstream alterations, leading to complex behavioral changes.

In the present study, higher concentrations of aluminum, copper and iron were found in AD patients compared with HCs. There is emerging evidence that elevated peripheral levels of aluminum may cause cognitive and motor deficits, as well as behavioral problems[16]. Our results suggest that aluminum is a major risk factor in AD. The evidence was derived to a large extent from our epidemiological studies that compared disease rates in subjects who were supplied with drugs and vessels containing different amounts of aluminum. Aluminum was available to humans through drinking water, aluminum vessels, aluminum foils used in food packing, and the higher levels in certain drugs, such as antacids[17]. The cerebrospinal fluid, BBB and nasal olfactory pathway are three routes through which aluminum could enter the brain from the systemic circulation[18]. The high concentration in plasma facilitates movement of aluminum to the brain, where it stimulates glutamate receptor activity and leads to an increased level of neuronal protein that is not bound by microtubules, and self-assembly of free leads to formation of paired helical filaments, which are the main component of NFTs[19].

The biochemistry of aging was a focus for understanding the etiology of AD. One of the primary hypotheses to account for this process is the oxidative damage induced by generation of ROS[20], and the damage is linked to the accumulation of redox-active copper or iron[21]. Hydrogen peroxide, one type of ROS, is freely permeable to the tissue membrane and increases Aβ neurotoxicity. If not scavenged by antioxidant enzymes like catalase and glutathione peroxidase, hydrogen peroxide could react with Cu+ or Fe2+ via the Fenton reaction to generate hydroxyl ions. In this situation, the cellular antioxidant defense mechanisms become overwhelmed by ROS production and exacerbate the toxicity of Aβ, while neurons appear to be particularly vulnerable to hydroxyl ion attack[22]. On the other hand, the liver incorporates copper into ceruloplasmin and secrete it into the plasma, and a slight defect in its liver regulation could have an effect on plasma copper concentration[23]. A few studies have reported data suggestive of abnormalities of copper incorporation into ceruloplasmin in AD patients. However, whether ceruloplasmin is a factor in AD and whether it is a factor in all AD cases is still debatable[24,25].

Iron is a redox active metal. In AD patients, iron increased in plasma also catalyzed the Fenton reaction, which gave rise to a flux of ROS that can potentially damage functional and structural macromolecules. Additionally, in some studies, iron appears to be involved in regulating the relationship between furin and α-secretases. Furin was ubiquitously expressed in many tissues[26]. Plasma levels of furin could be reflected in the brain and could enhance the activity of α-secretases[27], however, it is also modulated by iron concentration. α-Secretases cleave within the Aβ region, destroying the Aβ sequence and producing the neuroprotective soluble amyloid precursor protein α (sAPPα), which is a known trophic factor for neurons[28]. Once plasma iron concentration increases in AD, it can downregulate furin level, impairing the ability of α-secretases to produce sAPPα, and as a consequence, Aβ increases. In our study, the levels of furin were decreased and AUC was 0.914, as demonstrated by the ROC curve. This suggests that furin is an important biomarker identified in AD patients and stimulating furin activity or interfering with the pathways of iron regulation of furin could become a therapeutic target to increase production of the sAPPα neuroprotective form.

In sharp contrast, the plasma concentrations of lithium, manganese and zinc were decreased more in AD patients than HCs, and intriguingly, manganese was found to decrease twofold. It has been suggested that plasma manganese, similar to zinc, shows an inverse correlation with Aβ1–42 plaque load[29], and according to our work, the content of Aβ1–42 is increased in AD. On the other hand, the mitochondrial manganese superoxide dismutase (SOD) of the antioxidant defense systems that protect the brain from oxidative stress seems to play a key role in the onset of AD. SOD, localized in the mitochondrial membrane, is considered the primary defense against the superoxide radical and catalyzes the conversion of superoxide radicals to hydrogen peroxide, while catalase helps remove hydrogen peroxide formed during the reaction catalyzed by SOD. Plasma manganese tends to decrease in AD may weaken the ability of SOD to against the superoxide radical. In this situation, a cluster of molecular aberrations within the cell must increase Aβ1–42 neurotoxicity in the brain of AD patients. In addition, it is worth noting that iron overload can reduce manganese accumulation in the brain and other organs, since both of them share the same transport and regulatory proteins[30]. As we demonstrated, iron concentration was elevated in AD patients.

Zinc is released from the neocortical glutamatergic synapse, which is in communication with the plasma, and Aβ-trapped zinc accumulates in the neocortical glutamatergic synapse, which depletes zinc from other body compartments, such as plasma[31]. In other words, levels of plasma zinc may partly reflect circulation from synapses. This view was supported by a study in which plasma zinc level in AD patients was less than in HCs, however, after treatment with the zinc-binding compound clioquinol, plasma zinc returned to the normal range[32]. Clioquinol allows the synaptic zinc to resume its contribution to plasma by breaking down Aβ in the synapse. Therefore, decreased peripheral zinc may be a feature of AD. Furthermore, IDE and neprilysin are involved in the clearance of Aβ, and like furin, plasma IDE and neprilysin could also be reflected in the brain. Intriguingly, both of them have a catalytic site that contains a zinc ion. IDE comprises two domains, IDE-N and IDE-C, and the catalytic center is located in IDE-N, decreased plasma zinc concentrations can weak the expression of IDE that reduce the ability of IDE to clear Aβ. In conclusion, there are two situations that can decrease the ability of IDE to perform proteolysis of Aβ, boosting insulin levels and decreasing zinc concentrations[33]. Interestingly, the concomitant increase in insulin and Aβ levels may lead to redistribution of available IDE away from its function as an Aβ-degrading enzyme, which is why patients with type II diabetes have an increased risk of AD. However, the level of IDE was significantly lower in AD, it is still not an optimal biomarker for AD. In contrast, neprilysin, a thermolysin-like zinc metalloendopeptidase, plays an important role in degrading Aβ[34,35] and zinc coordination is necessary for its catalytic site with the conserved HExxH motif. In our study, there was no difference between the two groups and there was a lower AUC (<0.7) in the ROC curve. The debatable role of plasma neprilysin in the pathogenesis of AD needs further study.

Lithium has been demonstrated as effective in the treatment of mood disorder, especially bipolar disorder[36,37]. Few papers have been presented concerning the role of plasma lithium in AD. According to our study, great changes were observed in AD plasma concentrations of lithium. Lithium has a significant positive effect in synaptic plasticity and can reduce protein phosphorylation. We did not point out the role that lithium plays in the pathogenesis of AD. However, it is precisely because of this neuroprotective effect that lithium deficiency is a risk factor for AD and this requires further study.

In summary, 21 metals were measured simultaneously in plasma by ICP-MS. Multivariate statistical analysis defined intergroup differences between AD patients and HCs. ROC curve analysis was performed to compare the predictive ability of important biomarkers. All these analyses demonstrated that aberrations in metal homeostasis, as well as metalloproteinases, could be related to the development of AD. Manganese, aluminum, lithium, copper, iron and zinc showed the most significant changes, and could partly mediate synaptic dysfunction and neuronal toxicity. IDE, and furin, which have a strong correlation with zinc and iron, also play an important role in the etiology of AD and, we consider that plasma furin, may be a potential biomarkers in AD patients. Metallomics demonstrates the relationship between metals and AD. Further studies in this direction are currently ongoing in our laboratory.
